# Supplementary material for: Multi‐dataset identification of innovative feature genes and molecular mechanisms in keratoconus
Source: J Cell Mol Med. 2024 Sep 19;28(18):e70079. doi: 10.1111/jcmm.70079 (PMC11412914; doi:10.1111/jcmm.70079)
Supplement: Supplementary file 2 — Material S2. [file JCMM-28-e70079-s002.zip › 7.GSEA analysis/1.ARL11/KEGG_PHENYLALANINE_METABOLISM.html]

Details for gene set KEGG\_PHENYLALANINE\_METABOLISM[GSEA]

|  || Dataset | ARL11.group.cls#h\_versus\_l |
| Phenotype | group.cls#h\_versus\_l |
| Upregulated in class | h |
| GeneSet | KEGG\_PHENYLALANINE\_METABOLISM |
| Enrichment Score (ES) | 0.35709998 |
| Normalized Enrichment Score (NES) | 0.9851139 |
| Nominal p-value | 0.4940476 |
| FDR q-value | 1.0 |
| FWER p-Value | 1.0 |
Table: GSEA Results Summary

  

Fig 1: Enrichment plot: KEGG\_PHENYLALANINE\_METABOLISM      
 Profile of the Running ES Score & Positions of GeneSet Members on the Rank Ordered List

  

| SYMBOL | TITLE | RANK IN GENE LIST | RANK METRIC SCORE | RUNNING ES | CORE ENRICHMENT || 1 | MAOB | na | 1064 | 0.382 | 0.1685 | Yes |
| 2 | AOC2 | na | 1449 | 0.342 | 0.3355 | Yes |
| 3 | IL4I1 | na | 4200 | 0.194 | 0.3571 | Yes |
| 4 | GOT2 | na | 9613 | 0.066 | 0.2354 | No |
| 5 | PAH | na | 9882 | 0.062 | 0.2597 | No |
| 6 | ALDH3B1 | na | 11840 | 0.038 | 0.2230 | No |
| 7 | MAOA | na | 12281 | 0.034 | 0.2278 | No |
| 8 | ALDH3B2 | na | 13954 | 0.019 | 0.1896 | No |
| 9 | MIF | na | 18538 | -0.014 | 0.0648 | No |
| 10 | GOT1 | na | 19508 | -0.021 | 0.0476 | No |
| 11 | PRDX6 | na | 20232 | -0.025 | 0.0400 | No |
| 12 | ALDH3A1 | na | 20262 | -0.026 | 0.0526 | No |
| 13 | DDC | na | 23583 | -0.057 | -0.0134 | No |
| 14 | TAT | na | 24898 | -0.075 | -0.0124 | No |
| 15 | HPD | na | 27499 | -0.119 | -0.0255 | No |
| 16 | ALDH1A3 | na | 28596 | -0.146 | 0.0191 | No |
| 17 | AOC3 | na | 32726 | -0.302 | 0.0577 | No |
Table: GSEA details [plain text format]

  

Fig 2: KEGG\_PHENYLALANINE\_METABOLISM      
 Blue-Pink O' Gram in the Space of the Analyzed GeneSet

  

Fig 3: KEGG\_PHENYLALANINE\_METABOLISM: Random ES distribution      
 Gene set null distribution of ES for **KEGG\_PHENYLALANINE\_METABOLISM**
